# *Stenotrophomonas maltophilia* colonization during allogeneic hematopoietic stem cell transplantation is associated with impaired survival

**DOI:** 10.1371/journal.pone.0201169

**Published:** 2018-07-19

**Authors:** Sebastian Scheich, Rosalie Koenig, Anne C. Wilke, Sarah Lindner, Claudia Reinheimer, Thomas A. Wichelhaus, Michael Hogardt, Volkhard A. J. Kempf, Johanna Kessel, Sarah Weber, Hans Martin, Gesine Bug, Hubert Serve, Björn Steffen

**Affiliations:** 1 Department of Hematology and Oncology, Johann Wolfgang Goethe University of Frankfurt, Frankfurt am Main, Germany; 2 University Center for Infectious Diseases, University Hospital Frankfurt, Frankfurt am Main, Germany; 3 Institute of Medical Microbiology and Infection Control, University Hospital Frankfurt, Frankfurt am Main, Germany; 4 Department of Medicine, Infectious Diseases Unit, University Hospital Frankfurt, Frankfurt am Main, Germany; University of Kentucky, UNITED STATES

## Abstract

Allogeneic hematopoietic stem cell transplantation (allo-HSCT) offers potential cure to acute myeloid leukemia (AML) patients. However, infections with commensal bacteria are an important cause for non-relapse mortality (NRM). We have previously described the impact of multidrug-resistant organism (MDRO) colonization on the survival of allo-HSCT patients. In the aforementioned publication, according to consensus, we there did not consider the opportunistic gram-negative bacterium *Stenotrophomonas maltophilia* (*S*. *maltophilia*) to be an MDRO. Since rate of *S*. *maltophilia* colonization is increasing, and it is not known whether this poses a risk for allo-HSCT patients, we here analyzed here its effect on the previously described and now extended patient cohort. We report on 291 AML patients undergoing allo-HSCT. Twenty of 291 patients (6.9%) were colonized with *S*. *maltophilia*. Colonized patients did not differ from non-colonized patients with respect to their age, remission status before allo-HSCT, donor type and HSCT-comorbidity index. *S*. *maltophilia* colonized patients had a worse overall survival (OS) from 6 months up to 60 months (85% vs. 88.1% and 24.7% vs. 59.7%; p = 0.007) due to a higher NRM after allo-HSCT (6 months: 15% vs. 4.8% and 60 months: 40.1% vs. 16.2% p = 0.003). The main cause of mortality in colonized patients was infection (46.2% of all deaths) and in non-colonized patients relapse (58.8% of all deaths). 5/20 colonized patients developed an invasive infection with *S*. *maltophilia*. The worse OS after allo-HSCT due to higher infection related mortality might implicate the screening of allo-HSCT patients for *S*. *maltophilia* and a closer observation of colonized patients as outpatients.

## Introduction

Allogeneic hematopoietic stem cell transplantation (allo-HSCT) is a life-saving option for patients with advanced hematologic diseases like relapsed or refractory acute myeloid leukemia (AML). Often, it remains the only curative option. Over the last decade, improved transplant regimens lead to greatly reduced transplant-related morbidity and mortality. Therefore, the number of performed allo-HSCT continues to rise [1]. Graft-versus-Host disease (GvHD) and relapse of the underlying disease are considered the main causes of mortality during and after transplantation. However, the severe immunosuppression that accompanies allo-HSCT and GvHD prophylaxis and treatment puts patients at serious risk to suffer and die from infectious complications [2–7]. Aside from fungal and viral infections, bacterial infections, often with commensal bacteria, pose a considerable threat to these patients. A particularly menacing group of bacteria are gram-negative bacilli exhibiting a broad spectrum resistance to commonly used antibiotics like multidrug resistant *Enterobacteriaceae* and non-fermentative pathogens [8,9]. *Stenotrophomonas maltophilia* (*S*. *maltophilia*) is a widely spread gram negative opportunistic bacterial pathogen with increasing prevalence [9,8,10]. Due to low virulence it does not fit in the common definitions of multidrug-resistant organisms (MDRO)[11]. However, it has an intrinsic resistance against *e*.*g*. carbapenems [8,9]. Although its virulence is comparably low, *S*. *maltophilia* may cause severe infections especially in immunocompromised patients and patients on intensive-care units [9,8,10]. Once an infection occurs, treatment might be limited due to resistance to ß-lactam-antibiotics and increasing resistance rates to fluoroquinolones and cotrimoxazole [10,12–17]. Furthermore, underlying hematological diseases were shown to be an independent risk factor associated with a higher mortality of *S*. *maltophilia* infections [18].

This prompted us to revisit a previously analyzed and now extended patient cohort, in which we had analyzed the role of colonization with MDRO (but not *S*. *maltophilia)* after allo-HSCT [19]. Here, we have investigated the impact of *S*. *maltophilia* colonization on the outcome of AML patients post allo-HSCT. We hypothesize that colonization leads to clinically relevant infection throughout the course of immunosuppression impairing the survival of these patients.

## Materials, study design and definitions

We revisited the medical records of 264 patients with a diagnosis of AML, who underwent a first allo-HSCT at our institution between January 2006 and March 2016 and extended the cohort to December 2016 up to overall 291 patients. At our institution, all patients are routinely screened for colonization with multidrug-resistant organisms (namely Methicillin-resistant *Staphylococcus aureus*, Vancomycin-resistant *Enterococci*, gram-negative rods expressing extended-spectrum β-lactamase), and for *S*. *maltophilia* at the day of admittance and weekly thereafter by rectal, pharyngeal and nasal swabs.

Species identification and antibiotic susceptibility testing was performed as previously described [20]. All laboratory procedures were performed under quality–controlled criteria (laboratory accreditation according to ISO 15189:2007 standards; certificate number D–ML–13102–01–00, valid through January 25th, 2021). For *S*. *maltophilia* detection, swabs were collected using culture swabs with Amies collection and transport medium (Hain Lifescience, Nehren, Germany) and streaked onto selective gram-negative agar plates. Species were identified by biochemical identification systems or matrix-assisted–laser desorption ionization–time of flight analysis (API identification systems, VITEK MS, MALDI-TOF; bioMérieux, Nürtingen, Germany). Antibiotic susceptibility was tested according to Clinical and Laboratory Standards Institute (CLSI) guidelines using VITEK 2 and/or antibiotic gradient tests (bioMérieux) as well as agar diffusion method.

Colonization with *S*. *maltophilia* was defined as detection of the organism in at least one screening swab performed at the day of admittance and weekly thereafter during the hospital stay for allo-HSCT. Infection with *S*. *maltophilia* was defined as detection of the organism in blood culture bottles (BD BACTEC Lytic/10 Anaerobic/F and BD BACTEC Plus Aerobic/F, Becton Dickinson, Heidelberg, Germany) or primarily sterile body compartments together with clinical signs of infection.

For allo-HSCT, patients were individually housed in air-filtered rooms (HEPA-Filter) and transplants were performed according to local standard procedures with routinely inserted central venous catheters. According to our anti-infective guidelines, all patients received from the beginning of neutropenia until engraftment an antibiotic prophylaxis with cefotaxime and an anti-fungal prophylaxis containing an echinocandin or broad-spectrum azole. Additionally, all patients received cotrimoxazole three times a week for *Pneumocystis jirovecii* prophylaxis and acyclovir as long as CD4+ T-cells were <400/nl even during severe neutropenia. In case of fever, blood cultures were collected and bottles were sent for microbiological testing to the Institute for Medical Microbiology and Infection Control of University Hospital Frankfurt. Bloodstream infection was defined as detection of any bacterial species from blood cultures. For coagulase negative *staphylococci* two consecutive positive cultures were required to define an infection. According to Bacigalupo et al., conditioning regimens were classified as myeloablative (MAC)[21] or reduced intensity [22]. GvHD was assessed using modified Glucksberg criteria [23] (acute GvHD, aGvHD) or National Institute of Health criteria [24] (chronic GvHD, cGvHD), respectively. Mucositis was defined and graded according to Common Terminology Criteria for Adverse Events (CTCAE) [25]. Cytogenetic risk classification was done according to the guidelines of the European LeukemiaNet (ELN)[21]. Hematopoietic stem cell comorbidity index (HCT-CI) was used to assess pre-existing conditions [26]. The primary endpoints were overall survival (OS) and non-relapse related mortality (NRM), secondary endpoints were rate of neutropenic fever, infections with *S*. *maltophilia*, admission to intensive care unit and relapse of disease (>5% bone marrow blasts). Patients gave written informed consent for the use of their medical records and the study was approved by the Ethics Committee of the Medical Faculty of the Johann-Wolfgang Goethe University, Frankfurt, Germany (Approval number SHN-02-2017).

### Statistics

SPSS (Version 24.0; IBM, SPSS Institute Inc., Chicago, USA) and R (Version 3.2.2, packages “cmprsk” and “survival”) were used for statistical analysis. Comparisons of continuous variables were performed via by Mann-Whitney-U test and categorical variables via Fisher’s exact test and chi-square test, respectively. Kaplan-Meier curves were compared by log-rank test. For competing risks, cumulative incidences were calculated and compared using Gray’s test. For multivariate analysis, the Cox regression model was used with at least 10 events per variable according to Peduzzi et al. to include the variable into analysis [27].

## Results

### Baseline patient characteristics and Stenotrophomonas maltophilia findings

Between January 2006 and December 2016, 291 patients underwent a first allo-HSCT at the University Hospital Frankfurt, Germany and were included into the study. Table 1 shows baseline patient characteristics.

**Table 1:** Baseline patient characteristics. P-values reveals differences between colonized and non-colonized patients. Allo-HSCT, allogeneic hematopoietic stem cell transplantation; AML, acute myeloid leukemia; ELN, European Leukemia Net; CR, complete remission; MAC, myeloablative conditioning; PBSC, peripheral blood stem cells; MRD, matched related donor; MUD, matched unrelated donor; MMUD, mismatched unrelated donor; CMV, cytomegalovirus; GvHD, Graft versus Host Disease, HCT-CI, hemtaopoetic stem cell transplantation comorbidity index.

Characteristics	All patients (n = 291)	Non-colonized (n = 271)	Colonized (n = 20)	P-value
Year of allo-HSCT, median (range)	2012 (2006–2016)	2012 (2006–2016)	2009 (2006–2016)	0.050
Male sex, n (%)	173 (59.5)	160 (59)	13 (65)	0.646
Age at allo-HSCT, median (range)	54 (19–74)	53 (19–74)	57.5 (24–67)	0.303
De Novo AML, n (%)	195 (67)	184 (67.9)	11 (55)	0.324
**ELN cytogenetic risk, n (%)**				0.121
favorable	40 (13.7)	37 (13.7)	3 (15)	
Intermediate I/II	186 (63.9)	177 (65.3)	9 (45)	
adverse	65 (22.3)	57 (21)	8 (40)	
**Remission-status at allo-HSCT, n (%)**				0.340
CR 1	137 (47.1)	129 (47.6)	8 (40)	
≥CR2	35 (12)	34 (12.5)	1 (5)	
Active disease	119 (40.9)	108 (39.9)	11 (55)	
Conditioning MAC, n (%)	133 (45.7)	126 (46.5)	7 (35)	0.360
Graft type PBSC, n (%)	258 (88.7)	239 (88.2)	19 (95)	0.711
**Donor type**				0.802
MRD 10/10	76 (26.1)	70 (25.8)	6 (30)	
MUD 10/10	137 (47.1)	129 (47.6)	8 (40)	
MMUD 9/10 or 8/10	61 (21)	57 (21)	4 (20)	
Haploidentical	17 (5.8)	15 (5.5)	2 (10)	
CMV Recipient+/Donor-, n (%)	46 (15.8)	43 (15.9)	3 (15)	1.000
AB0-Mismatch, n (%)	157 (54)	145 (53.5)	12 (60)	0.648
GvHD prophylaxis: ATG containing, n (%)	217 (74.6)	204 (75.3)	13 (65)	0.298
Months from diagnosis/relapse to allo-HSCT, median (range)	3.07 (0.27–38.27)	3.07 (0.27–15.97)	3.28 (1.13–38.27)	0.930
**HCT-CI**				0.780
Low risk, n (%)	69 (23.7)	64 (23.6)	5 (25)	
Intermediate risk, n (%)	93 (32)	88 (32.5)	5 (25)	
High risk, n (%)	129 (44.3)	119 (43.9)	10 (50)	

The median age of the study population was 54 years (range 19–74) being slightly predominated by male patients (173/291, 59.5%). Most patients were transplanted in first remission (137/291, 47.1%), 35 (12%) in second or later remission and 119/291 (40.9%) with active disease. 67% of patients had de novo AML with 13.7% favorable, 63.9% intermediate I/II and 22.3% adverse ELN cytogenetic risk score. 133/291 patients (45.7%) received MAC conditioning and 88.7% were peripheral blood grafts. 26.1% were transplanted from a matched related donor, 47.1% from a matched unrelated donor, 21% from a mismatched unrelated donor and 5.8% from a haploidentical donor. 217/291 patients received antithymocyte globulin (ATG) as GvHD prophylaxis. A high risk CMV constellation (recipient positive, donor negative) was found in 15.8% of patients, 54% were transplanted with an AB0 mismatch. The median time from diagnosis or relapse to transplantation was 3.07 months (range 0.27–38.27). Regarding pre-existing conditions, 23.7% had a low risk, 32% an intermediate risk and 44.3% a high risk HCT-CI.

In our study, 20/291 patients (6.9%) were colonized by *S*. *maltophilia*. Most of these colonized patients were colonized orally (16/20, 80%), two patients each (10%) had rectal colonization and nasal colonization, respectively. Concerning positive status for *S*. *maltophilia*, 18/20 patients (90%) were initially tested positive during their stay for allo-HSCT, 2/20 patients (10%) were reported to be positive before admission. The median time between admission to allo-HSCT and the first positive swab was 19.5 days (range 0–87 days). Resistance to fluoroquinolones (ciprofloxacin/levofloxacin) was found in 2/20 (10%), resistance to cotrimoxazole in 3/20 (15%) *S*. *maltophilia* colonized patients and a resistance against ceftazidime in 11/20 patients (55%). The median year of transplantation tends to be earlier for colonized patients compared to non-colonized patients (2009 vs. 2012, p = 0.050). Other baseline patient characteristics did not differ between colonized and non-colonized patients.

### Transplant-characteristics, events and outcomes

An overview of transplant-characteristics and outcomes is given in Table 2.

**Table 2:** Transplant-related characteristics and outcomes. P-values reveal differences between colonized and non-colonized patients. ANC, absolute neutrophil count; PLT, platelets; aGvHD, acute Graft versus Host Disease; cGvHD, chronic Graft versus Host Disease MDRO, multidrug resistant organisms; BIS, bloodstream infection; allo-HSCT, allogeneic hematopoietic stem cell transplantation. OS, Overall survival, 95% CI, 95% confidence interval; NRM, non-relapse mortality; GvHD, Graft versus Host Disease.

Characteristics	All patients (n = 291)	Non-colonized (n = 271)	Colonized (n = 20)	P-value
Engraftment ANC > 0.5 × 10^9^/l (days), median (range)	18 (9–36)	18 (10–36)	16 (9–28)	0.077
Engraftment PLT > 50 × 10^9^/l (days), median (range)	19 (9–1575)	19.5 (9–1575)	19 (14–67)	0.306
Length of hospital stay (days), median (range)	42 (7–180)	41 (7–180)	52.5 (31–153)	0.011
Neutropenic fever, n (%)	245 (84.2)	226 (83.4)	19 (95)	0.218
*C*. *diff* toxin positive, n (%)	49 (16.8)	44 (16.2)	5 (25)	0.350
Mucositis grade 3/4, n (%)	124 (42.6)	115 (42.4)	9 (45)	0.819
Cumulative incidence of aGvHD, % (95% CI)	50.5 (44.7, 56.3)	49.6 (43.5, 55.7)	63.2 (40.5, 85.9)	0.202
Cumulative incidence of aGvHD grade 3/4, % (95% CI)	10.2 (6.7, 13.7)	9.5 (6, 13)	21.1 (2.2, 40)	0.099
Cumulative incidence of cGvHD, % (95% CI)	47.9 (41.7, 54.1)	47.7 (41.3, 54.1)	50 (25.6, 74.4)	0.664
Cumulative incidence of severe cGvHD, % (95% CI)	12.1 (8.1, 16.1)	11.8 (7.7, 15.9)	16.7 (0, 34.7)	0.558
Intensive care unit stay, n (%)	18 (6.2)	13 (4.8)	5 (25)	0.004
Bloodstream infections, n (%)	88 (30.2)	79 (29.2)	9 (45)	0.205
*S*. *maltophilia* infection, n (%)	6 (2.1)	1 (0.4)	5 (25)	<0.001
*S*. *maltophilia* BSI infection, n (%)	2 (0.7)	0	2 (10)	0.005
CMV-reactivation, n (%)	N = 201 112 (55.7)	N = 187 107 (57.2)	N = 14 5 (35.7)	0.163
Other viral infection during allo-HSCT, n (%)	84 (28.9)	78 (28.8)	6 (30)	1.000
**Estimated OS, % (95% CI)**				0.007
6 months OS	87.9 (84.2, 91.6)	88.1(84.2, 92)	85 (69.3, 100)	
12 months OS	80.8 (76.3, 85.3)	81.6 (76.9, 86.3)	69.1(48.5, 89.7)	
48 months OS	60 (53.7, 66.3)	62.1(55.6, 68.6)	33 (8.5, 57.5)	
60 months OS	57 (50.3, 63.7)	59.7 (53, 66.4)	24.7 (1.6, 47.8)	
**Cumulative incidence of NRM, % (95% CI)**				0.003
6 months OS	5.5 (3.4, 7.6)	4.8 (2.2, 7.4)	15 (0, 31.1)	
12 months OS	9.4 (6, 12.8)	8.2 (4.9, 11.5)	25 (5.4, 44.6)	
48 months OS	17 (12.1, 21.9)	15.3 (10.5, 20.1)	40.1 (14.4, 65.8)	
60 months OS	17.9 (12.8, 23)	16.2 (11.1, 21.3)	40.1(14.4, 65.8)	
5-year Cumulative incidence of relapse, % (95% CI)	34.6 (28.2, 41)	34.2 (27.6, 40.8)	37.9 (8.7, 67.1)	0.927
**Death caused by, n (%/% of all deaths)**				0.021
infection	29 (10/9.1)	23 (8.5/23.7)	6 (30/46.2)	
relapse	62 (21.3/56.4)	57 (21/58.8)	5 (25/38.5)	
GvHD	7 (2.4/6.4)	7 (2.6/7.2)	0	
others	11(3.8/10)	10 (3.7/10.3)	1 (5/7.7)	
unknown	1 (0.3/0.9)	0	1 (5/7.7)	
**Death in neutropenia after allo-HSCT, n (%)**	8 (2.8)	6 (2.2)	2 (10)	0.098

The median length of the hospital stay for allo-HSCT was 42 days (range 7–180) with a median neutrophil engraftment time (>0.5×10^9^/l) of 18 days (range 9–36) and a median platelet engraftment time (>50×10^9^/l) of 19 days (range 9–1575). Neutrophil engraftment time tends to be shorter in colonized patients (16 days vs. 18 days, p = 0.077). Neutropenic fever occurred in 84.2% of patients and 16.8% were tested positive for *Clostridium difficile*. 124/291 patients (42.6%) suffered from a mucositis CTCAE grade 3/4 and in 88/291 (30.2%) a blood stream infection (BSI) was detected. CMV-reactivation was observed in 55.7% of all patients (with donor and/or recipient CMV-positive; n = 201), other viral infections in 28.9% of all patients during allo-HSCT. In case of fever during allo-HSCT, carbapenems were the most commonly used antibiotics (65.3%), followed by piperacillin/tazobactam (31.3%). 30.2% of all patients received fluoroquinolones (ciprofloxacin or levofloxacin). Colistin was administered in 4.8%, amikacin in 12%, vancomycin in 35.7%, linezolid in 24.7%, teicoplanin in 16.2% and tigecyclin in 1% of all patients. 18 patients had to be admitted to an intensive care unit (ICU) during their stay for allo-HSCT. Patients colonized with *S*. *maltophilia* had a significantly longer inpatient stay for allo-HSCT (52.5 days vs. 41 days, p = 0.011) and were more often admitted to ICU (5/20 patients, 25% vs. 13/271 patients, 4.8%, p = 0.004). 80% (4/5 patients) of the colonized patients were admitted to ICU because of respiratory insufficiency and 7/13 (54.8%) patients from the non-colonized group were admitted to the ICU due to respiratory insufficiency. Six patients suffered from an infection by *S*. *maltophilia* (two pulmonary infections, one each with wound infection, urinary tract infection, BSI and combined pulmonary and BSI, respectively). More colonized patients suffered from a *S*. *maltophilia* infection than patients tested negative for *S*. *maltophilia* (25% vs. 0.4%, p<0.001). Interestingly, one patient without prior colonization primarily presented with an urinary tract infection by a *S*. *maltophilia* infection.

The cumulative incidence of acute GvHD (aGvHD) was 50.5% (95% CI 44.7, 56.3) with a grade 3 or 4 GvHD incidence of 10.2% (95% CI 6.7, 13.7). The cumulative incidence of chronic GvHD (cGvHD) was 47.9% (95% CI 41.7, 54.1) and the severe cGvHD rate was 12.1% (95% CI 8.1, 16.1). Cumulative incidences of aGvHD, grade 3 or 4 aGvHD, cGvHD and severe cGvHD did not differ between non-colonized patients and patients tested positive for *S*. *maltophilia*.

**Fig 1:**
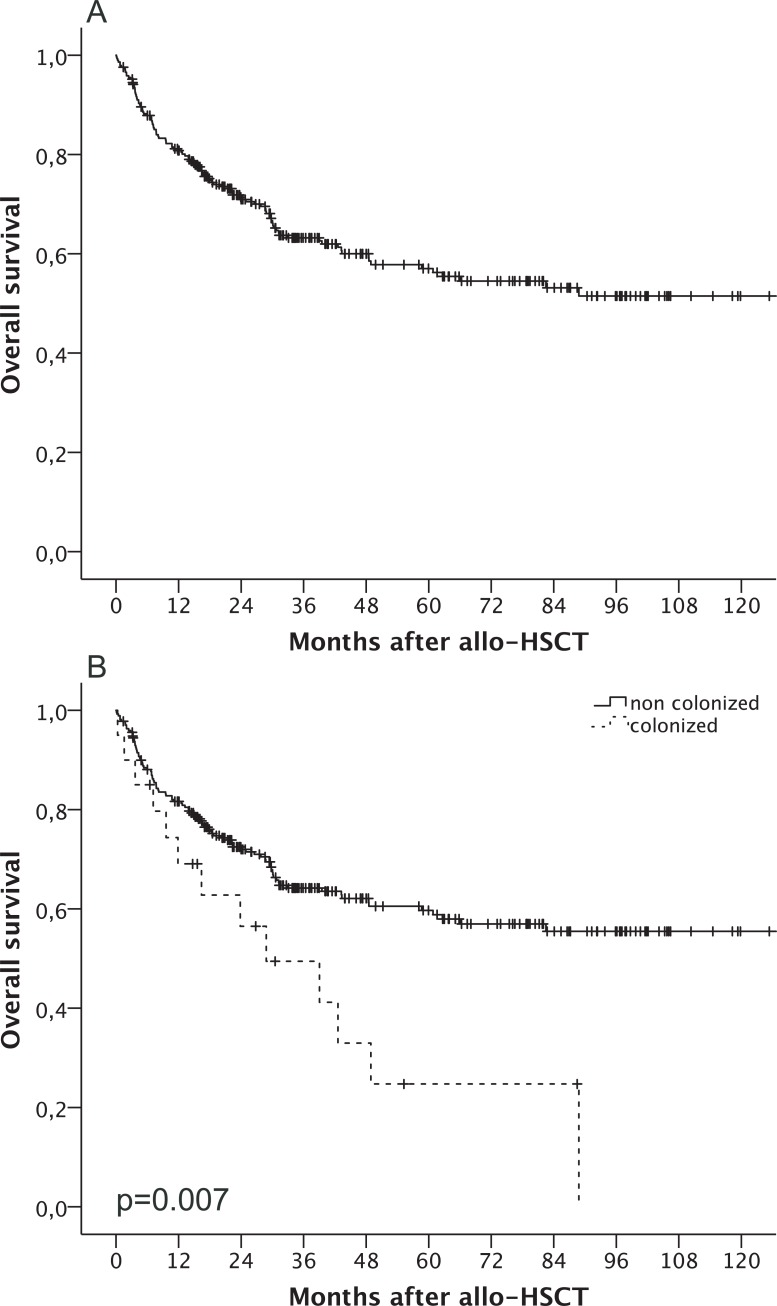
Overall survival after allogeneic hematopoietic stem cell transplantation. (A) Overall survival of all patients. (B) Overall survival stratified by colonization (dotted line) with *S*. *maltophilia*.

The estimated 5-year OS of all patients was 57% with a mean OS time of 84.6 months (95% CI 76.5, 92.7; Fig 1A). Colonized patients revealed a lower OS after allo-HSCT compared to non-colonized patients (p = 0.007): OS was 88.1% (95% CI, 84.2, 92) in non-colonized and 85% (95% CI 69.3, 100) in colonized patients at 6 months, 81.6% (95% CI 76.9, 86.3) in non-colonized and 69.1% (95% CI 48.5, 89.7) in colonized patients at 12 months, 62.1% (95% CI 55.6, 68.6) in non-colonized and 33% (95% CI 8.5, 57.5) in colonized patients at 48 months and 59.7% (95% CI 53, 66.4) in non-colonized and 24.7% (95% CI 1.6, 47.8) in colonized patients after 60 months (Fig 1B).

**Fig 2:**
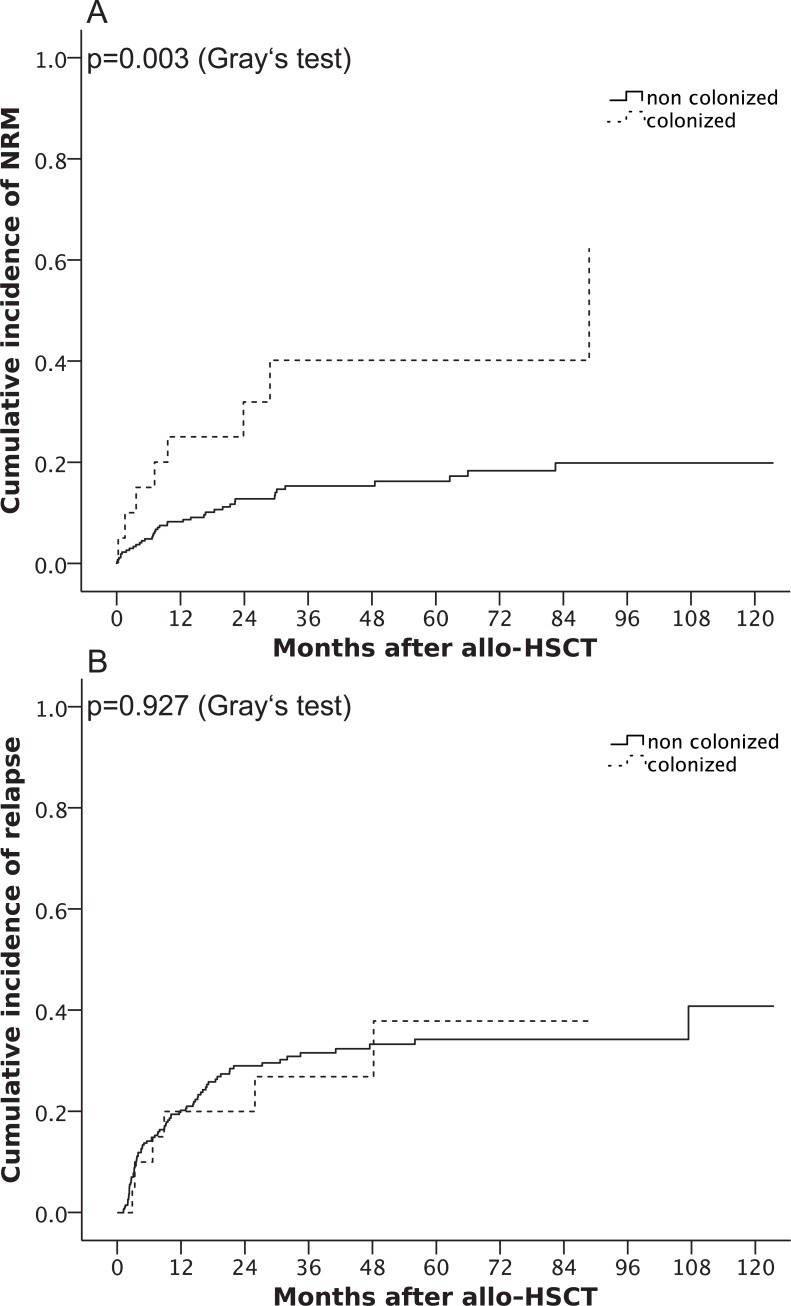
**Cumulative incidence of non-relapse related mortality (A) and cumulative incidence of relapse (B)** after allogeneic hematopoietic stem cell transplantation, stratified by colonized (dotted line) and non-colonized (solid line) patients.

Lower OS in the colonized group was mainly attributable to a higher cumulative incidence of NRM. NRM rose from 4.8% (95% CI 2.2, 7.4) in non-colonized patients and 15% (95% CI 0, 31.1) in colonized patients at 6 months up to a 5-year cumulative incidence of NRM of 16.2% (95% CI 11.1, 21.3) in non-colonized and 40.1% (95% CI 14.4, 65.8) in colonized patients (p = 0.003; Fig 2A). The cumulative incidence of relapse (5 years) did not differ significantly between both groups (34.2% in non-colonized vs. 37.9% in colonized patients, p = 0.927; Fig 2B).

Regarding the causes of death, infection was the main cause in colonized patients (46.2% of all deaths), while in non-colonized patients, relapse of AML was the most frequent cause of fatal outcome (56.4% of all deaths, p = 0.021). All deaths in colonized patients were attributable to lung infections. Infection related deaths of colonized patients are described in detail in the supplementary Table 1 (S1 Table). 2.8% of all patients (8/291) died in neutropenia immediately after allo-HSCT, 6 (2.2%) from the non-colonized and 2 (10%) from colonized group (p = 0.098).

Multivariate analysis of OS (Table 3) revealed that colonization with *S*. *maltophilia* (hazard ratio (HR) 1.982, 95% CI 1.091–3.597; p = 0.025) and adverse ELN-cytogenetics vs. favorable cytogenetics (HR 2.319, 95% CI 1.138–4.726; p = 0.021) were independent risk factors for fatal outcome, whereas year of transplantation ≥2012, ELN-cytogenetics, which were both differing in univariate analysis, and age >60 were not.

**Table 3:** Multivariate analysis for overall survival. 95% CI, 95% confidence interval; allo-HSCT, allo-HSCT, allogeneic hematopoietic stem cell transplantation; ELN, European Leukemia Net. Of all *S*.*maltophilia* colonized patients (n = 20), 15 (75%) were also colonized by an MDRO as described previously[19].

Risk Factor	Hazard ratio	95% CI	P-value
*Stenotrophomas maltophilia-colonization*	1.982	1.091–3.597	0.025
Year of allo-HSCT ≥2012	1.059	0.715–1.569	0.775
Intermediate ELN (vs. favorable)	1.392	0.712–2.722	0.334
Adverse ELN (vs. favorable)	2.319	1.138–4.726	0.021
Age > 60	1.276	0.847–1.921	0.243

In an exploratory multivariate analysis for OS including only colonization with MDRO and *S*. *maltophilia*, *S*. *maltophilia* was identified as an independent risk factor for death (MDRO colonization: HR 1.734, 95% CI 1.159–2.595, p = 0.007; *S*. *maltophilia* colonization: HR 1.879, 95% CI 1.045–3.381, p = 0.035). In comparison to non-colonized patients (neither colonized with *S*. *maltophilia* nor with MDRO), patients colonized with *S*. *maltophilia* had an inferior OS (S1 Fig) compared to non-colonized patients (5-year OS: 24.7% vs. 66.3%; p = 0.001) and compared to MDRO-colonized patients (5-year OS: 24.7% vs. 53.3%; p = 0.065).

## Discussion

Patients with AML undergoing allo-HSCT are at a particular high risk of developing infections with *S*. *maltophilia* due to chemotherapy, disease related immunosuppression, severe neutropenia and inserted central venous catheters [28–30]. Therefore, we have reviewed clinical records of 291 AML patients, who underwent a first allo-HSCT at our institution to clarify the role of colonization with *S*. *maltophilia* on outcome parameters and have identified 20 (6.9%) patients who were colonized swab by *S*. *maltophilia* in at least one.

In the original, not extended cohort (264 patients) we have recently shown that MDRO-positive patients had an inferior OS probability compared to non-colonized patients at 5 years primarily due to a higher cumulative incidence of NRM after allo-HSCT[19]. In this study, of 20 patients colonized by *S*. *maltophilia*, 15 were additionally colonized by a MDRO which may confound our results. However, the explorative multivariate analysis for OS including only MDRO-colonization and *S*. *maltophilia* colonization revealed that *S*. *maltophilia* colonization is an independent risk factor for fatal outcome.

The colonization rate of 6.9% (20 patients) is comparable to other studies: Shiratori et al. reported from a colonization rate of 6.4% (14/220 patients)[31]. In our study, colonized patients revealed in our study a significant lower OS in univariate analysis as well as in multivariate analysis (HR 1.982, 95% CI 1.091–3.597; p = 0.025) due to a higher NRM compared to non-colonized patients (5-year cumulative incidence of NRM: 40.1% vs. 16.2%, p = 0.003). The main causes of mortality were infection (46.2% of all deaths) in colonized and relapse (56.4% of all deaths) in non-colonized patients. It is well known from MDRO (*e*.*g*. VRE, MRSA or multidrug resistant gram-negative bacteria) that colonization is an important risk factor for developing an infection with the respective pathogen [32,33]. We found that 5/20 colonized patients (25%) developed an infection with *S*. *maltophilia*. An appropriate causal chain of colonization with *S*. *maltophilia*, severe infection with *S*. *maltophilia* and immediate death due to *S*. *maltophilia* has been found for one patient, who died directly attributable to a *S*. *maltophilia* BSI. In our study, 10% of all colonized patients died during neutropenia after allo-HSCT as inpatients due to infections whereas this was the case for only 2.2% of non-colonized patients died during neutropenia infection related after allo-HSCT (p = 0.098). Furthermore, colonized patients were admitted more frequently to the ICU (25% vs. 4.8%, p = 0.004) due to pulmonary infections (4/5 patients were transferred to the ICU due to pulmonary insufficiency). Though it is known that ICU stay is a risk factor for *S*. *maltophilia* infections [34,35,18], all five patients, which were transferred to the ICU in our study were colonized before their ICU admission. Interestingly, median time between admission and the first detection of *S*. *maltophilia* was 19.5 days (range 0–87 days), which represents the time of aGvHD (immediately after neutrophil engraftment), but the rates of aGvHD revealed no differences between colonized and non-colonized patients. Surprisingly, in addition to the slightly higher inpatient mortality, main differences in NRM between colonized and non-colonized patients appeared after 6 months due to infections and differences in OS appeared after 12 months.

There are three possible explanations for impaired OS as an outpatient in the colonized group: (i) colonized patients may die due to invasive infections with *S*. *maltophilia* but the low rate of broncho-alveolar lavages at our institution and the low sensitivity of culture techniques consequently lead to the missing proof of *S*. *maltophilia* infections. This explanation might be supported by the fact, that all colonized patients with fatal infections as an outpatient (n = 6, 56.4% of all deaths in colonized group) suffered from pulmonary infections. In 4/6 patients *S*. *maltophilia* was detected orally immediately before their death and *S*. *maltophilia* regularly cause lung infections in immunocompromised patients with high mortality [36] (ii) Other infections in the outpatient period (when patients left close clinical monitoring) occur more frequently in colonized patients. The immunomodulatory effects of *S*. *maltophilia* are well described [37] which may favor other infections. So, colonization with *S*. *maltophilia* might be a marker for a not fully hosted immune response after allo-HSCT and patients might die related to infections due to *S*. *maltophilia* immune dysregulation. (iii) *S*. *maltophilia* colonization might be a surrogate marker for medical unfit patients (or other unknown conditions), even if no differences in HCT-CI score could be found, *e*.*g*. with pre-existing lung diseases leading to later fatal outcome in the colonized group since all of the fatal infections in colonized patients were lung infections.

In our study, 2.1% (6/291) of all patients suffered from invasive infections caused by *S*. *maltophilia*. Other studies reported an infection rate of *S*. *maltophilia* of 5.6% (16/287 patients)[38] or 10.7% (25/234 patients)[31] in the setting of allo-HSCT, which is much higher than in our study. The difference might be due to the frequent usage of fluoroquinolones in colonized patients in 55% (11/20) of all cases at our center (30.2% of all patients received fluoroquinolones) and a susceptibility of *S*. *maltophilia* to these antibiotics in most of the patients in our cohort (18/20; 90%). The major advantage of our study is the large and homogenous AML patient cohort. Most of the baseline patient characteristics did not differ between the two groups and differing baseline characteristics (year of transplantation) did not influence the negative impact of *S*. *maltophilia* colonization on OS in multivariate analysis. It is especially important, that aGvHD and cGvHD cumulative incidences did not differ between both groups, because GvHD is one of the major risk factors for NRM after allo-HSCT [39,40].

We take into account that this is a retrospective study, direct causal conclusions can be drawn only to limited extend. Furthermore, the group of colonized patients consists of only 20 patients, therefore our results have to be interpreted carefully. It remains inconclusive if *S*. *maltophilia* causes infections leading to death in the period after transplantation or if it is a surrogate marker for an inadequate immune response or other unknown factors. We cannot fully address this question due to the small number of events in the colonized group and larger further prospective studies are needed.

We conclude that colonization with *S*. *maltophilia* is associated with an impaired OS after allo-HSCT due to higher rates of infection-related deaths. This might indicate to monitor colonized patients closely as outpatients due to a possibly increased susceptibility to infections. Further prospective studies are required to elucidate reasons of higher infection rates.

## Supporting information

S1 Table Reasons for infection-related death in colonized patients. (DOCX)

S1 Fig Overall survival after allogeneic hematopoietic stem cell transplantation stratified for non-colonized, MDRO colonized and *S*. *maltophilia* colonized patients. (DOCX)
