# Supplementary material for: Stenotrophomonas maltophilia colonization during allogeneic hematopoietic stem cell transplantation is associated with impaired survival
Source: PLoS One. 2018 Jul 19;13(7):e0201169. doi: 10.1371/journal.pone.0201169 (PMC6053200; doi:10.1371/journal.pone.0201169)
Supplement: S1 Table — (DOCX) [file pone.0201169.s001.docx]

| ***S. maltophilia* colonized patients** |
| --- |
| H1N1 pneumonia |
| Mucor pneumoniae and lung bleeding |
| Pneumonia, bloodstream infection with *Staphylococcus xylosus*; oral *S. maltophilia* detection immediately before death |
| Aspergillus pneumonia oral and rectal *S. maltophilia* detection immediately before death |
| Pneumonia and bloodstream infection with *Pseudomonas aeruginosa*; oral *S. maltophilia* detection immediately before death |
| Pneumonia and oral *S. maltophilia* detection and *S. maltophilia* bloodstream infection immediately before death |

**Reasons for infection-related death in colonized patients.**
