# Supplementary material for: Stenotrophomonas maltophilia colonization during allogeneic hematopoietic stem cell transplantation is associated with impaired survival
Source: PLoS One. 2018 Jul 19;13(7):e0201169. doi: 10.1371/journal.pone.0201169 (PMC6053200; doi:10.1371/journal.pone.0201169)
Supplement: S1 Fig — (DOCX) [file pone.0201169.s002.docx]

**Supporting figure 1. Reasons for infection-related death in colonized patients.**
